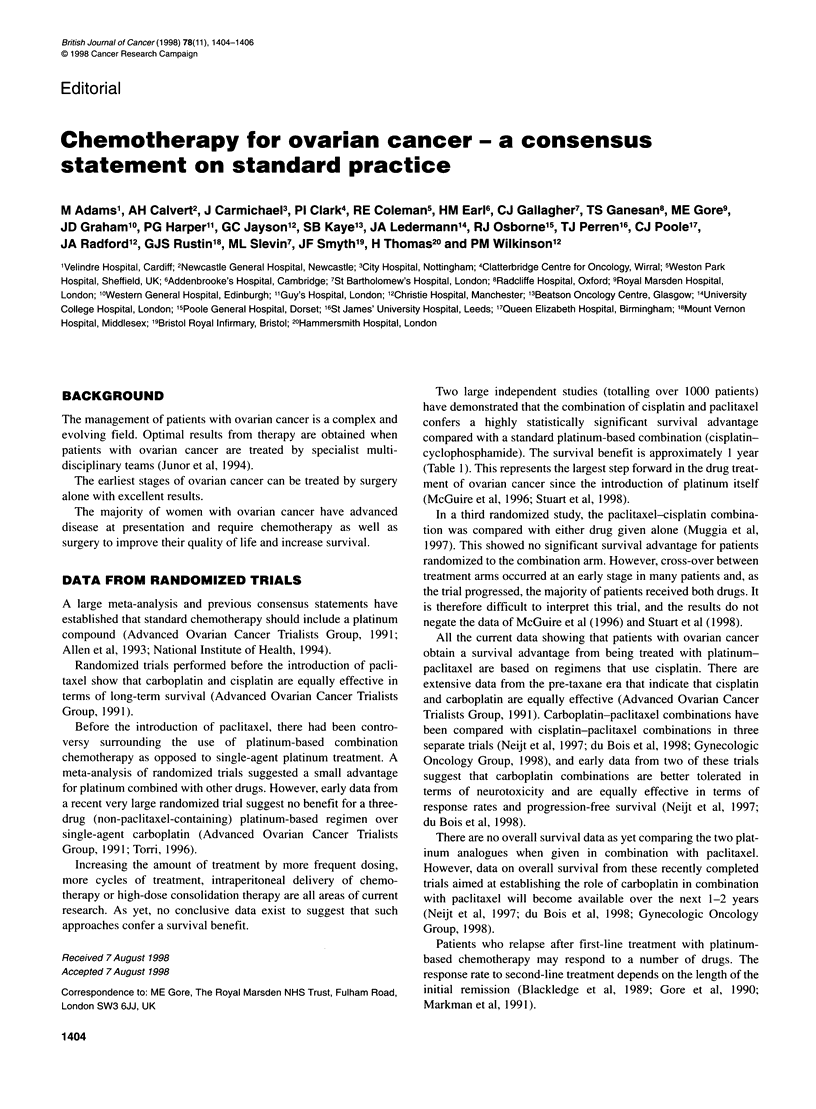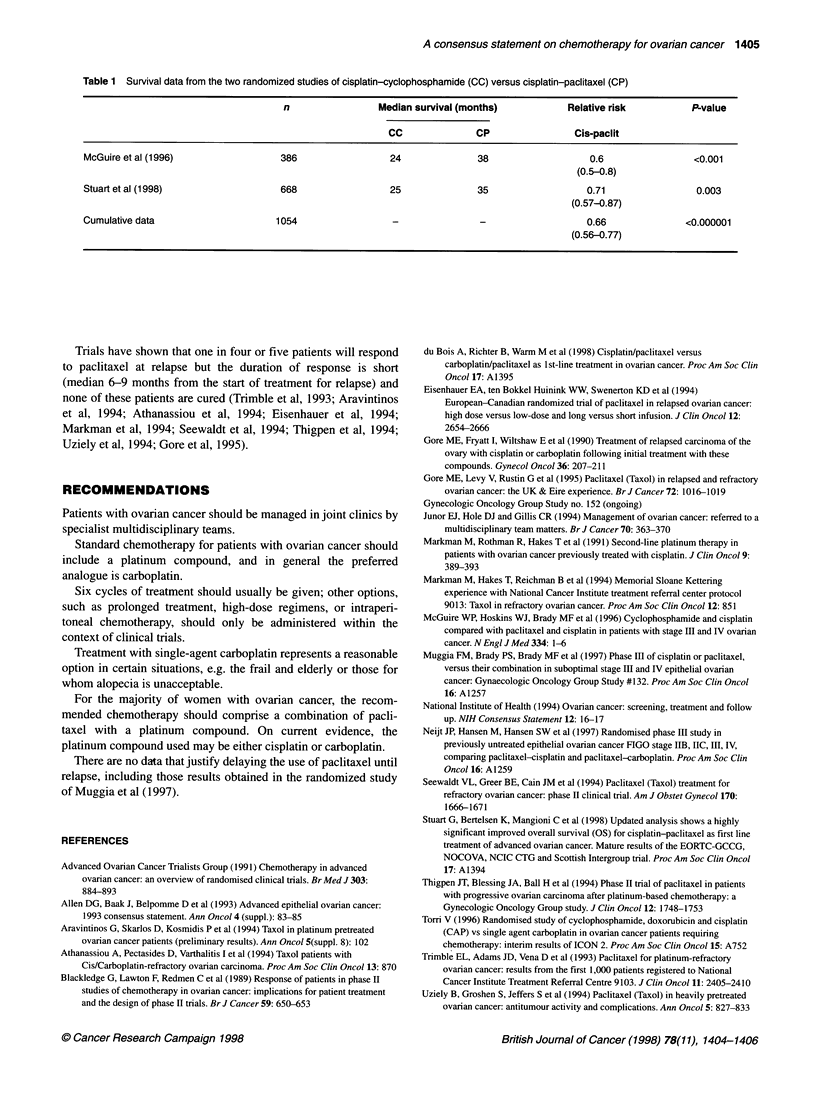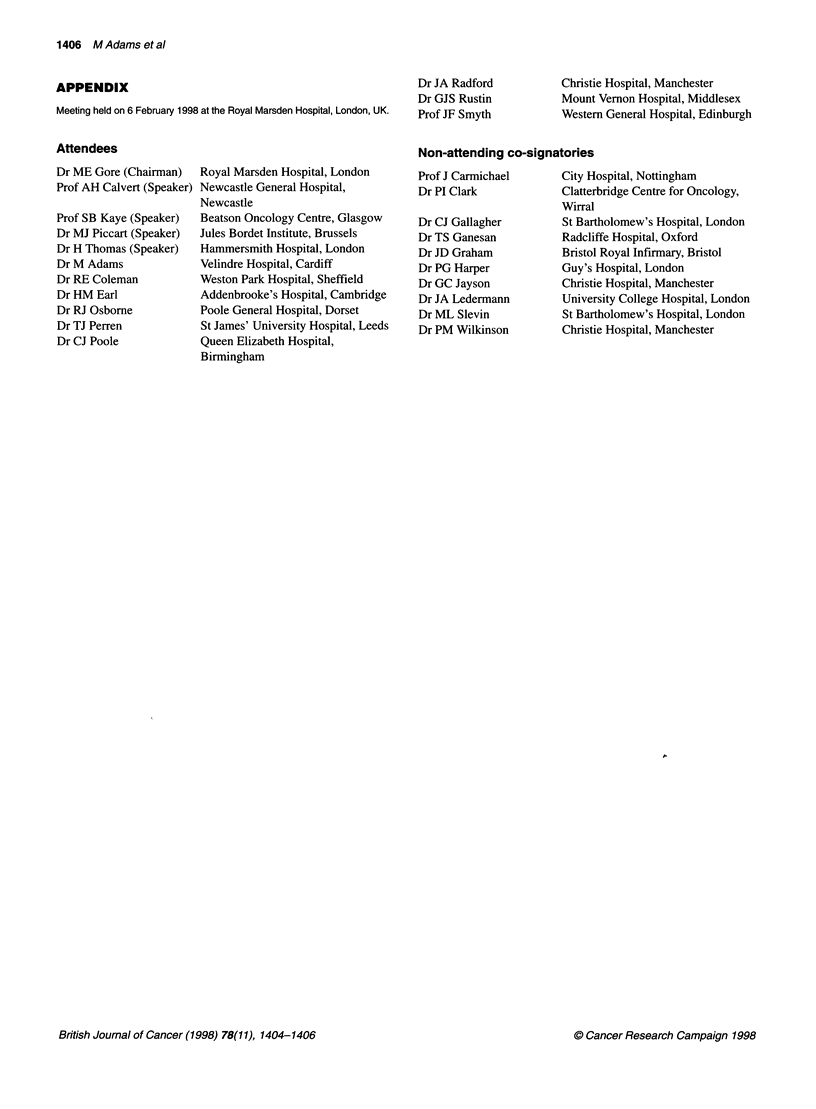# Chemotherapy for ovarian cancer--a consensus statement on standard practice.

**DOI:** 10.1038/bjc.1998.699

**Published:** 1998-12

**Authors:** M. Adams, R. P. A'Hern, A. H. Calvert, J. Carmichael, P. I. Clark, R. E. Coleman, H. M. Earl, C. J. Gallagher, T. S. Ganesan, M. E. Gore, J. D. Graham, P. G. Harper, G. C. Jayson, S. B. Kaye, J. A. Ledermann, R. J. Osborne, T. J. Perren, C. J. Poole, J. A. Radford, G. J. Rustin, M. L. Slevin, J. F. Smyth, H. Thomas, P. M. Wilkinson


British Journal of Cancer (1998) 78(11), 1404–1406
© 1998 Cancer Research Campaign

Editorial

### Chemotherapy for ovarian cancer – a consensus statement on standard practice

**M Adams[1], AH Calvert[2], J Carmichael[3], PI Clark[4], RE Coleman[5], HM Earl[6], CJ Gallagher[7], TS Ganesan[8], ME Gore[9], JD Graham[10], PG Harper[11], GC Jayson[12], SB Kaye[13], JA Ledermann[14], RJ Osborne[15], TJ Perren[16], CJ Poole[17], JA Radford[12], GJS Rustin[18], ML Slevin[7], JF Smyth[19], H Thomas[20] and PM Wilkinson[12]**

[1]Velindre Hospital, Cardiff; [2]Newcastle General Hospital, Newcastle; [3]City Hospital, Nottingham; [4]Clatterbridge Centre for Oncology, Wirral; [5]Weston Park Hospital, Sheffield, UK; [6]Addenbrooke's Hospital, Cambridge; [7]St Bartholomew's Hospital, London; [8]Radcliffe Hospital, Oxford; [9]Royal Marsden Hospital, London; [10]Western General Hospital, Edinburgh; [11]Guy's Hospital, London; [12]Christie Hospital, Manchester; [13]Beatson Oncology Centre, Glasgow; [14]University College Hospital, London; [15]Poole General Hospital, Dorset; [16]St James' University Hospital, Leeds; [17]Queen Elizabeth Hospital, Birmingham; [18]Mount Vernon Hospital, Middlesex; [19]Bristol Royal Infirmary, Bristol; [20]Hammersmith Hospital, London

### BACKGROUND

The management of patients with ovarian cancer is a complex and evolving field. Optimal results from therapy are obtained when patients with ovarian cancer are treated by specialist multi-disciplinary teams (Junor et al, 1994).

The earliest stages of ovarian cancer can be treated by surgery alone with excellent results.

The majority of women with ovarian cancer have advanced disease at presentation and require chemotherapy as well as surgery to improve their quality of life and increase survival.

### DATA FROM RANDOMIZED TRIALS

A large meta-analysis and previous consensus statements have established that standard chemotherapy should include a platinum compound (Advanced Ovarian Cancer Trialists Group, 1991; Allen et al, 1993; National Institute of Health, 1994).

Randomized trials performed before the introduction of paclitaxel show that carboplatin and cisplatin are equally effective in terms of long-term survival (Advanced Ovarian Cancer Trialists Group, 1991).

Before the introduction of paclitaxel, there had been controversy surrounding the use of platinum-based combination chemotherapy as opposed to single-agent platinum treatment. A meta-analysis of randomized trials suggested a small advantage for platinum combined with other drugs. However, early data from a recent very large randomized trial suggest no benefit for a three-drug (non-paclitaxel-containing) platinum-based regimen over single-agent carboplatin (Advanced Ovarian Cancer Trialists Group, 1991; Torri, 1996).

Increasing the amount of treatment by more frequent dosing, more cycles of treatment, intraperitoneal delivery of chemotherapy or high-dose consolidation therapy are all areas of current research. As yet, no conclusive data exist to suggest that such approaches confer a survival benefit.

*Received 7 August 1998*
*Accepted 7 August 1998*

Correspondence to: ME Gore, The Royal Marsden NHS Trust, Fulham Road, London SW3 6JJ, UK

Two large independent studies (totalling over 1000 patients) have demonstrated that the combination of cisplatin and paclitaxel confers a highly statistically significant survival advantage compared with a standard platinum-based combination (cisplatin–cyclophosphamide). The survival benefit is approximately 1 year (Table 1). This represents the largest step forward in the drug treatment of ovarian cancer since the introduction of platinum itself (McGuire et al, 1996; Stuart et al, 1998).

In a third randomized study, the paclitaxel–cisplatin combination was compared with either drug given alone (Muggia et al, 1997). This showed no significant survival advantage for patients randomized to the combination arm. However, cross-over between treatment arms occurred at an early stage in many patients and, as the trial progressed, the majority of patients received both drugs. It is therefore difficult to interpret this trial, and the results do not negate the data of McGuire et al (1996) and Stuart et al (1998).

All the current data showing that patients with ovarian cancer obtain a survival advantage from being treated with platinum–paclitaxel are based on regimens that use cisplatin. There are extensive data from the pre-taxane era that indicate that cisplatin and carboplatin are equally effective (Advanced Ovarian Cancer Trialists Group, 1991). Carboplatin–paclitaxel combinations have been compared with cisplatin–paclitaxel combinations in three separate trials (Neijt et al, 1997; du Bois et al, 1998; Gynecologic Oncology Group, 1998), and early data from two of these trials suggest that carboplatin combinations are better tolerated in terms of neurotoxicity and are equally effective in terms of response rates and progression-free survival (Neijt et al, 1997; du Bois et al, 1998).

There are no overall survival data as yet comparing the two platinum analogues when given in combination with paclitaxel. However, data on overall survival from these recently completed trials aimed at establishing the role of carboplatin in combination with paclitaxel will become available over the next 1–2 years (Neijt et al, 1997; du Bois et al, 1998; Gynecologic Oncology Group, 1998).

Patients who relapse after first-line treatment with platinum-based chemotherapy may respond to a number of drugs. The response rate to second-line treatment depends on the length of the initial remission (Blackledge et al, 1989; Gore et al, 1990; Markman et al, 1991).

**Table 1** Survival data from the two randomized studies of cisplatin–cyclophosphamide (CC) versus cisplatin–paclitaxel (CP)

| | *n* | Median survival (months) | | Relative risk | *P*-value |
|---|---|---|---|---|---|
| | | CC | CP | Cis-paclit | |
| McGuire et al (1996) | 386 | 24 | 38 | 0.6 (0.5–0.8) | <0.001 |
| Stuart et al (1998) | 668 | 25 | 35 | 0.71 (0.57–0.87) | 0.003 |
| Cumulative data | 1054 | – | – | 0.66 (0.56–0.77) | <0.000001 |

Trials have shown that one in four or five patients will respond to paclitaxel at relapse but the duration of response is short (median 6–9 months from the start of treatment for relapse) and none of these patients are cured (Trimble et al, 1993; Aravintinos et al, 1994; Athanassiou et al, 1994; Eisenhauer et al, 1994; Markman et al, 1994; Seewaldt et al, 1994; Thigpen et al, 1994; Uziely et al, 1994; Gore et al, 1995).

### RECOMMENDATIONS

Patients with ovarian cancer should be managed in joint clinics by specialist multidisciplinary teams.

Standard chemotherapy for patients with ovarian cancer should include a platinum compound, and in general the preferred analogue is carboplatin.

Six cycles of treatment should usually be given; other options, such as prolonged treatment, high-dose regimens, or intraperitoneal chemotherapy, should only be administered within the context of clinical trials.

Treatment with single-agent carboplatin represents a reasonable option in certain situations, e.g. the frail and elderly or those for whom alopecia is unacceptable.

For the majority of women with ovarian cancer, the recommended chemotherapy should comprise a combination of paclitaxel with a platinum compound. On current evidence, the platinum compound used may be either cisplatin or carboplatin.

There are no data that justify delaying the use of paclitaxel until relapse, including those results obtained in the randomized study of Muggia et al (1997).

### REFERENCES

Advanced Ovarian Cancer Trialists Group (1991) Chemotherapy in advanced ovarian cancer: an overview of randomised clinical trials. *Br Med J* **303**: 884–893

Allen DG, Baak J, Belpomme D et al (1993) Advanced epithelial ovarian cancer: 1993 consensus statement. *Ann Oncol* **4** (suppl.): 83–85

Aravintinos G, Skarlos D, Kosmidis P et al (1994) Taxol in platinum pretreated ovarian cancer patients (preliminary results). *Ann Oncol* **5**(suppl. 8): 102

Athanassiou A, Pectasides D, Varthalitis I et al (1994) Taxol patients with Cis/Carboplatin-refractory ovarian carcinoma. *Proc Am Soc Clin Oncol* **13**: 870

Blackledge G, Lawton F, Redmen C et al (1989) Response of patients in phase II studies of chemotherapy in ovarian cancer: implications for patient treatment and the design of phase II trials. *Br J Cancer* **59**: 650–653

du Bois A, Richter B, Warm M et al (1998) Cisplatin/paclitaxel versus carboplatin/paclitaxel as 1st-line treatment in ovarian cancer. *Proc Am Soc Clin Oncol* **17**: A1395

Eisenhauer EA, ten Bokkel Huinink WW, Swenerton KD et al (1994) European–Canadian randomized trial of paclitaxel in relapsed ovarian cancer: high dose versus low-dose and long versus short infusion. *J Clin Oncol* **12**: 2654–2666

Gore ME, Fryatt I, Wiltshaw E et al (1990) Treatment of relapsed carcinoma of the ovary with cisplatin or carboplatin following initial treatment with these compounds. *Gynecol Oncol* **36**: 207–211

Gore ME, Levy V, Rustin G et al (1995) Paclitaxel (Taxol) in relapsed and refractory ovarian cancer: the UK & Eire experience. *Br J Cancer* **72**: 1016–1019

Gynecologic Oncology Group Study no. 152 (ongoing)

Junor EJ, Hole DJ and Gillis CR (1994) Management of ovarian cancer: referred to a multidisciplinary team matters. *Br J Cancer* **70**: 363–370

Markman M, Rothman R, Hakes T et al (1991) Second-line platinum therapy in patients with ovarian cancer previously treated with cisplatin. *J Clin Oncol* **9**: 389–393

Markman M, Hakes T, Reichman B et al (1994) Memorial Sloane Kettering experience with National Cancer Institute treatment referral center protocol 9013: Taxol in refractory ovarian cancer. *Proc Am Soc Clin Oncol* **12**: 851

McGuire WP, Hoskins WJ, Brady MF et al (1996) Cyclophosphamide and cisplatin compared with paclitaxel and cisplatin in patients with stage III and IV ovarian cancer. *N Engl J Med* **334**: 1–6

Muggia FM, Brady PS, Brady MF et al (1997) Phase III of cisplatin or paclitaxel, versus their combination in suboptimal stage III and IV epithelial ovarian cancer: Gynaecologic Oncology Group Study #132. *Proc Am Soc Clin Oncol* **16**: A1257

National Institute of Health (1994) Ovarian cancer: screening, treatment and follow up. *NIH Consensus Statement* **12**: 16–17

Neijt JP, Hansen M, Hansen SW et al (1997) Randomised phase III study in previously untreated epithelial ovarian cancer FIGO stage IIB, IIC, III, IV, comparing paclitaxel–cisplatin and paclitaxel–carboplatin. *Proc Am Soc Clin Oncol* **16**: A1259

Seewaldt VL, Greer BE, Cain JM et al (1994) Paclitaxel (Taxol) treatment for refractory ovarian cancer: phase II clinical trial. *Am J Obstet Gynecol* **170**: 1666–1671

Stuart G, Bertelsen K, Mangioni C et al (1998) Updated analysis shows a highly significant improved overall survival (OS) for cisplatin–paclitaxel as first line treatment of advanced ovarian cancer. Mature results of the EORTC-GCCG, NOCOVA, NCIC CTG and Scottish Intergroup trial. *Proc Am Soc Clin Oncol* **17**: A1394

Thigpen JT, Blessing JA, Ball H et al (1994) Phase II trial of paclitaxel in patients with progressive ovarian carcinoma after platinum-based chemotherapy: a Gynecologic Oncology Group study. *J Clin Oncol* **12**: 1748–1753

Torri V (1996) Randomised study of cyclophosphamide, doxorubicin and cisplatin (CAP) vs single agent carboplatin in ovarian cancer patients requiring chemotherapy: interim results of ICON 2. *Proc Am Soc Clin Oncol* **15**: A752

Trimble EL, Adams JD, Vena D et al (1993) Paclitaxel for platinum-refractory ovarian cancer: results from the first 1,000 patients registered to National Cancer Institute Treatment Referral Centre 9103. *J Clin Oncol* **11**: 2405–2410

Uziely B, Groshen S, Jeffers S et al (1994) Paclitaxel (Taxol) in heavily pretreated ovarian cancer: antitumour activity and complications. *Ann Oncol* **5**: 827–833

© Cancer Research Campaign 1998

### APPENDIX

Meeting held on 6 February 1998 at the Royal Marsden Hospital, London, UK.

### Attendees

| | |
|---|---|
| Dr ME Gore (Chairman) | Royal Marsden Hospital, London |
| Prof AH Calvert (Speaker) | Newcastle General Hospital, Newcastle |
| Prof SB Kaye (Speaker) | Beatson Oncology Centre, Glasgow |
| Dr MJ Piccart (Speaker) | Jules Bordet Institute, Brussels |
| Dr H Thomas (Speaker) | Hammersmith Hospital, London |
| Dr M Adams | Velindre Hospital, Cardiff |
| Dr RE Coleman | Weston Park Hospital, Sheffield |
| Dr HM Earl | Addenbrooke's Hospital, Cambridge |
| Dr RJ Osborne | Poole General Hospital, Dorset |
| Dr TJ Perren | St James' University Hospital, Leeds |
| Dr CJ Poole | Queen Elizabeth Hospital, Birmingham |
| Dr JA Radford | Christie Hospital, Manchester |
| Dr GJS Rustin | Mount Vernon Hospital, Middlesex |
| Prof JF Smyth | Western General Hospital, Edinburgh |

### Non-attending co-signatories

| | |
|---|---|
| Prof J Carmichael | City Hospital, Nottingham |
| Dr PI Clark | Clatterbridge Centre for Oncology, Wirral |
| Dr CJ Gallagher | St Bartholomew's Hospital, London |
| Dr TS Ganesan | Radcliffe Hospital, Oxford |
| Dr JD Graham | Bristol Royal Infirmary, Bristol |
| Dr PG Harper | Guy's Hospital, London |
| Dr GC Jayson | Christie Hospital, Manchester |
| Dr JA Ledermann | University College Hospital, London |
| Dr ML Slevin | St Bartholomew's Hospital, London |
| Dr PM Wilkinson | Christie Hospital, Manchester |

*© Cancer Research Campaign 1998*